# The Ionomics of Lettuce Infected by *Xanthomonas campestris* pv. *vitians*

**DOI:** 10.3389/fpls.2019.00351

**Published:** 2019-03-22

**Authors:** Olbert Nicolas, Marie Thérèse Charles, Sylvie Jenni, Vicky Toussaint, Serge-Étienne Parent, Carole Beaulieu

**Affiliations:** ^1^Département de Biologie, Université de Sherbrooke, Sherbrooke, QC, Canada; ^2^Agriculture and Agri-Food Canada, Saint-Jean-sur-Richelieu Research and Development Centre, Saint-Jean-sur-Richelieu, QC, Canada; ^3^Department of Soils and Agri-Food Engineering, Université Laval, Quebec City, QC, Canada

**Keywords:** bacterial leaf spot, ICP-OES, ionomics, *Lactuca sativa*, lettuce, nutrient balance, plant resistance, *Xanthomonas campestris*

## Abstract

Bacterial leaf spot (BLS) caused by *Xanthomonas campestris* pv. *vitians* (*Xcv*) places a major constraint on lettuce production worldwide. The most sustainable strategy known to date for controlling BLS is the use of resistant cultivars. The nutrient elemental signature (ionome) of ten lettuce cultivars with three levels of resistance was analyzed by inductively coupled plasma optical emission spectroscopy (ICP-OES) to determine which nutrient balances are linked to resistance to BLS, and to assess the effect of *Xcv* infection on the ionome. The elemental concentrations were preprocessed with isometric log-ratios to define nutrient balances. Using this approach, 4 out of 11 univariate nutrient balances were found to significantly influence the resistance of lettuce cultivars to BLS (*P* < 0.05). These significant balances were the overall nutritional status balancing all measured nutrients with their complementary in the dry mass, as well as balances [Mn | Zn,Cu], [Zn | Cu], and [S,N | P]. Moreover, the infection of lettuce cultivars mostly affected the lettuce ionome on the [N,S | P] balance, where infection tended to lean the balance toward the N,S part relatively to P. This study shows that nutrient uptake in lettuce can be affected by BLS infection and that nutrient status influences resistance to BLS infection.

## Introduction

Lettuce (*Lactuca sativa* L.) is one of the most popular vegetables worldwide. It is mostly cultivated in temperate and subtropical climates. With seven main groups of cultivars, lettuce is morphologically the most diverse species of the genus *Lactuca* as distinguished by phenotypic characteristics: crisphead, cos (romaine), butterhead, leaf, latin, stem, and oilseed (De Vries, 1997).

According to Lebeda et al. (2007), several diseases of lettuce have been described, but only a few are important enough to be considered in crop protection. Among these diseases, bacterial leaf spot (BLS) of lettuce caused by *Xanthomonas campestris* pv. *vitians* (*Xcv*) is economically important worldwide (Lu and Raid, 2013). BLS infection of lettuce is favored by warm and humid climatic conditions (Wang et al., 2015). Typical symptoms are black, water-soaked lesions on the leaves (Hayes et al., 2014), which may coalesce under conditions favorable to *Xcv* development, causing large necrotic spots on the leaves (Toussaint, 1999). The unsightly leaf blemishes render lettuce unmarketable (Robinson et al., 2006).

The current phytosanitary products used to control BLS have to date shown poor efficiency (Lu and Raid, 2013). Common control strategies such as prophylactic measures, specifically removal of crop debris and use of clean farm machinery, are often ineffective because occurrence of this disease is sporadic (Bull et al., 2015). Nevertheless, the most widely accepted strategy for controlling BLS of lettuce is the use of resistant cultivars (Bull and Koike, 2005). Therefore, sustainable management of disease resistance requires sound knowledge of factors that may contribute to this resistance. Different levels of resistance to BLS have been reported in lettuce, and the single dominant *Xanthomonas* resistance gene 1 (*Xar1*) has been identified as being responsible for resistance in some lettuce cultivars (Hayes et al., 2014). Other factors such as stomatal density of the leaves have been also reported to play a role in lettuce resistance (Nicolas et al., 2018).

Mineral elements play an important nutritional role in plants. Seventeen macronutrients (including C, H, O, N, S, P, Ca, K, and Mg) and micronutrients or trace nutrients (including Ni, Mo, Cu, Zn, Mn, B, Fe, and Cl) are known to be essential to all plants (Williams and Salt, 2009). Nutrients can affect the development of a disease by affecting plant physiology, the causal pathogen, or both (Dordas, 2009). Micronutrients are involved in diverse cellular functions, including energy metabolism, primary and secondary metabolism, defense, gene regulation, hormone perception, signal transduction, and reproduction (Hänsch and Mendel, 2009). High metal levels can restrict host colonization by microbial pathogens and be utilized as a defense mechanism by the host during plant-microbe interactions (Navarrete and De La Fuente, 2015).

The ionome, i.e., the “mineral nutrient and trace elements found in an organism” (Lahner et al., 2003), has been studied in many fields of biology, namely in agronomy (Jaradat and Goldstein, 2018), physiology (Baxter et al., 2008), ecology (Aerts and Chapin, 2000), and functional genetics (White and Brown, 2010). Lettuce contains several macro elements (e.g., Ca, Mg, Na, K) and trace elements (e.g., Fe, Mn, Zn, Cu) (Pinto et al., 2014). Several of these elements, by virtue of their biological functions, affect pathogenic interactions between bacteria and plants. Some are involved in biologic functions of the cell such as enzyme activation, regulation of gene expression, hormone synthesis and perception (DalCorso et al., 2014). The concentrations of transition metals in the environment and the availability of essential metals to support pathogen growth can have a significant impact on the outcome of plant-pathogen interactions (Fones and Preston, 2013).

Ionomes belong to the class of compositional data, i.e., data representing parts of a whole (Aitchison, 1986). The isometric log ratio (ilr) transformation overcomes inherent biases emerging from statistical analyses of compositional data by transforming components to non-overlapping orthonormal ratios of components (Egozcue et al., 2003). The ilr transformation is suitable for conducting multivariate analyses of ionomes, because it can illustrate the nutrient relationships as a multidimensionnal map of sound, hierarchically arranged binary balances betweeen groups of nutrients (Parent et al., 2013; Modesto et al., 2014). Nutrient balances can also be included in non-linear models to achieve greater accuracy (Silverman et al., 2017).

Considering that uptake and translocation of mineral elements in plants is influenced by plant species (Pinto et al., 2014), the objective of this study was to determine the following: (i) a possible relationship between the ionome and cultivar resistance and (ii) the effect of BLS infection on the ionome.

This study provides seminal information that could be used not only to develop a suitable fertilization strategy for the management of BLS in lettuce but also to improve understanding of the role played by nutrient balances in plant-pathogen interactions.

## Materials and Methods

### Plant Production

The lettuce cultivars used in this study were previously characterized for their resistance to BLS by Nicolas et al. (2018; Table 1), according to a modified version of the method developed by Bull et al. (2015). Cultivars were classified into three groups based on their susceptibility to BLS: tolerant group with Batavia Reine des Glaces (BRG), Little Gem (LIG), Estival (EST) and Hochelaga (HOC); intermediate group with Romora (ROM) and Turbo (TUR); and susceptible group with Chief (CHI), Gorilla (GOR), Paris Island Cos (PIC) and Vista Verde (VIV). These cultivars are representative lettuce types from the Agriculture and Agri-Food Canada lettuce breeding program (Nicolas et al., 2018).

**Table 1:** Susceptibility of the ten lettuce cultivars used in this study when inoculated with *Xanthomonas campestris* pv. *vitians* (after Nicolas et al., 2018).

Cultivar	Type	Plant ID^a^	Resistance to BLS
Chief	Romaine	PI635108	Susceptible
Gorilla	Romaine	PI630943	Susceptible
Paris Island Cos	Romaine	PI536817	Susceptible
Vista Verde	Romaine	W629938	Susceptible
Romora	Romaine	–^b^	Intermediate
Turbo	Romaine	–	Intermediate
Batavia R.G.	Batavia	PI634668	Tolerant
Hochelaga	Crisphead Great Lakes	–	Tolerant
Estival	Crisphead Vanguard	PI651883	Tolerant
Little Gem	Latin	PI617959	Tolerant

^a^*Plant ID is based on the United States National Plant Germplasm system.*

^b^*Information not available*.

Lettuce seeds were sown in seedling trays containing peat moss (Promix, Saint-Remi, QC, Canada) as growth substrate. The seedlings were placed in a growth chamber with the following conditions: temperature of 18°C (day) and 16°C (night), with 70% relative humidity and a 16-h photoperiod. After 21 days, the seedlings were transplanted into 15-cm-diameter pots, filled with the same growth substrate. Experimental units of lettuce were grown in a greenhouse with temperatures ranging from 14 to 28°C, 70% relative humidity and 16-h photoperiod. Plants were fertilized every week with a 20-8-20 (N-P-K) soluble fertilizer.

### Lettuce Inoculation

One week after transplantation, lettuce plants were infected with the rifampicin-resistant *X. campestris* pv. *vitians* strain B07-007, isolated from field-grown lettuce in the Montérégie region (QC, Canada). The bacterium was conserved at -80°C in a solution of glycerol and nutrient broth (50:50 vol:vol) and grown at 28°C in yeast extract-dextrose carbonate (YDC) agar for 72 h. Bacteria from the incubation plates were scraped off and suspended in saline buffer (8.5 g L^-1^ NaCl). The culture was diluted to a final concentration of 10^8^ colony-forming units (cfu) per mL (OD_600_ = 0.1) in saline buffer. Using an airbrush, the bacterial suspension was sprayed onto 28-day-old lettuces so that it would run off the abaxial and adaxial sides of the plant leaves. Control plants were sprayed with saline buffer only, and all plants were incubated in a greenhouse misting room with a mist set to spray fine water droplets for 30 s every 30 min for 12 h (6:00 AM to 6:00 PM) over 14 days. A total of 240 lettuces divided into 10 cultivars, 2 treatments, and 4 replicates were distributed in a randomized complete block design. The experiment was repeated three times independently.

### Ionome Characterization

The characterization was performed on 6-week-old lettuces. Fourteen days after infection, the whole plants, excluding the roots, were sampled. For each sample, three lettuces were placed in the same paper bag and dried at 70°C for 72 h. The dried plants were crushed into a fine powder, and a 5-g portion was used for further analysis. A total of 40 uninfected and 40 infected samples were prepared and used in the analyses. After digestion (Havlin and Soltanpour, 1980) of 1 g of dry sample with nitric acid, the analysis was performed for Ca, Cu, Fe, K, Mg, Mn, Na, P, S, and Zn by inductively coupled plasma optical emission spectroscopy (ICP-OES) using a Perkin Elmer Optima 3200DVom. The determination of total N in the lettuce tissues was performed following the method proposed by Issac and Johnson (1976).

### Statistical Analysis

The leaf ionome was preprocessed by transforming nutrient concentrations to balances of nutrients with the ilr technique. Isometric log ratios are computed as:

$$ilr_{i}\;=\;\sqrt{\frac{n_{i}^{+}\;n_{i}^{-}}{n_{i}^{+}\;+\;n_{i}^{-}}}ln\left(\frac{g\;\left(c_{i}^{+}\right)}{g\;\left(c_{i}^{-}\right)}\right)$$

where, in the *i^th^* row of the SBP, *n_i_^+^* and *n_i_^-^* are the numbers of components in the numerator group and the denominator group, respectively, *g(c_i_^+^)* is the geometric mean of components in the numerator group and *g(c_i_^-^)* is the geometric mean of components in the denominator group. Balances are noted [A,B | C,D], where components A and B at the denominator are balanced with components C and D at the numerator. A positive balance means that the geometric mean of concentrations of components C and D is larger than the geometric mean of concentrations of components A and B, conversely for a negative balance and even for a null balance. Hence, in linear modeling, a positive effect on [A,B | C,D] means that the increase of the importance of C and D compared to A and B is associated with an increases the response variable of the model.

**Figure 1:**
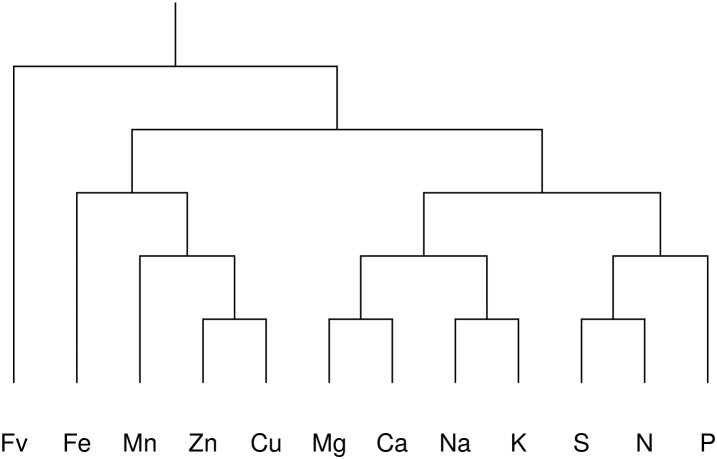
Bifurcating tree identification showing 11 hierarchical balances representing a subcomposition of nutrients in the lettuce ionome: [Fv | Fe,Mn,Zn,Cu,Mg,Ca,Na,K,S,N,P], [Fe, Mn,Zn,Cu | Mg,Ca,Na,K,S,N,P], [Fe | Mn,Zn,Cu], [Na,Mg,K,Ca | N,S,P], [Mn | Zn,Cu], [Mg,Ca | Na,K], [S,N | P], [Zn | Cu], [Mg | Ca], [Na | K], [S | N]. The experimental effect on nutrient balances was removed prior to data processing using mixed modeling with the nutrient balance as response and the experiment as random intercept (more details in the *Results* section).

The selected balance design for this study is presented in Figure 1. The filling value, Fv, is the amalgamation of all elements excluded from the analysis (mainly C, O, and H), computed by difference between the unit or scale of measurement and the sum of included elements.

Computations were performed in the R statistical language version 3.4.1 (R Development Core Team, 2017). The main packages used in the data analysis workflow were the vegan package version 2.4-3 (Oksanen et al., 2017) for ordination, the compositions package version 1.40-1 (van den Boogaart and Tolosana-Delgado, 2013) for ilr transformations, the nlme version 3.1-131 (Pinheiro et al., 2017) package to compute the random experimental effect, the mvoutlier package version 2.0.8 (Filzmoser and Gschwandtner, 2017) for multivariate outlier detection, and the ggplot2 package version 2.2.1 (Wickham and Chang, 2017) for data visualization. The data and computations are publicly available at https://github.com/essicolo/Nicolas-et-al_Infected-lettuce-ionomics.

## Results

### Data Preprocessing

Fourteen days post inoculation, symptoms were observed on the lettuce leaves according to their degree of resistance to BLS as described in Nicolas et al. (2018).

**Figure 2:**
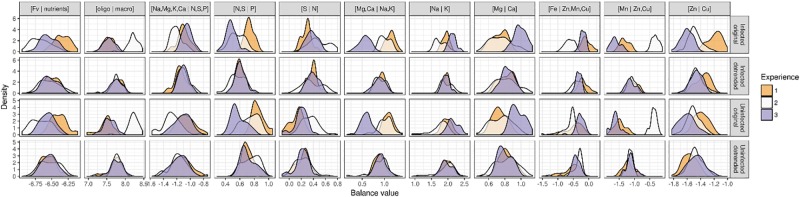
Density curves of balance variables by experiment and treatment before and after detrending the experimental effect.

Data exploration revealed a large experimental effect on the ionome, as shown in the density curves in Figure 2. A discriminant analysis (not shown) confirmed the density discrepancies. The second experiment stands apart from the first and third experiments mainly due to the [Mn | Zn,Cu] balance, the [Zn | Cu] balance and the [Fv | nutrients] balance. The three experiments differ mainly in terms of the [N,S | P] balance, the [Mg,Ca | Na,K] balance, and the [Zn | Cu] balance. Na is rather a beneficial element analyzed here as macronutrient as >1000 mg kg^-1^ lettuce dry weight (Pilon-Smith et al., 2009).

The experimental random effect was computed by applying a mixed model to each nutrient balance, with interacting cultivar, and treatment (infected or uninfected) as fixed effects and experiment as random intercept effect. This allowed us to compute a random effect on each balance in terms of offsets from the first experiment. After filtering out experimental effects by removing the offset from balance variables, we obtained data centered on the first experiment (Figure 2) and allowed comparisons of measured features. Using a permissive criterion for outlier detection, we excluded eight observations where unusual multivariate ionomic balances were detected.

### Effects of Cultivars and Treatments on the Ilr Values

**Figure 3:**
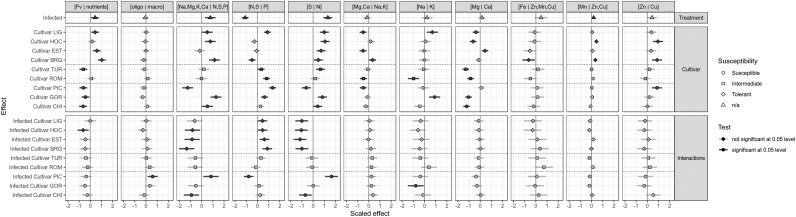
Effects of cultivars and treatments on the ilr values.

Figure 3 presents the effects of cultivars and treatments on scaled and centered ilr values. The effects are computed as linear coefficients of a mixed model with the experimental random effect. The susceptible cultivar VIV and the uninfected treatment were used as the reference categories. All effects should therefore be interpreted as a departure from the uninfected VIV. The infection itself significantly increased the proportion of measured nutrients, as shown by the positive effect of infection on the [Fv | nutrients] balance (Supplementary Table S1). While the [oligo | macro], [Mg,Ca | Na,K], [Na | K], [Mg | Ca], [Fe | Mn,Zn,Cu], and [Zn | Cu] balances showed almost no change, infection with BLS had a substantial effect on balances closely related to nitrogen, with a significant decrease in [N,S | P] and a significant increase in [Na,Mg,K,Ca | N,S,P] and [S | N]. The [Mn | Zn,Cu] balance showed a slight but significant increase due to the infection alone.

Cultivars also had different ionome trends, regardless of the treatment (Cultivar section in Figure 3). It should be emphasized that the effect is considered significant when a significant difference is found between the ionomic balance of a given cultivar and that of VIV. Significance can also be assessed on pairwise comparison by checking if the confidence intervals of the effects overlap. It can be observed from Figure 3 that the [Fv | nutrients], [Na,Mg,K,Ca | N,S,P], [N,S | P], and [Mg | Ca] balances differed markedly for the ionomes of the uninfected cultivars. The [Fv | nutrients] balance differed significantly between the tolerant cultivars and the susceptible ones, with all the susceptible cultivars showing a negative [Fv | nutrients] balance, and the tolerant cultivars, except HOC, showing a positive [Fv | nutrients] balance.

**Figure 4:**
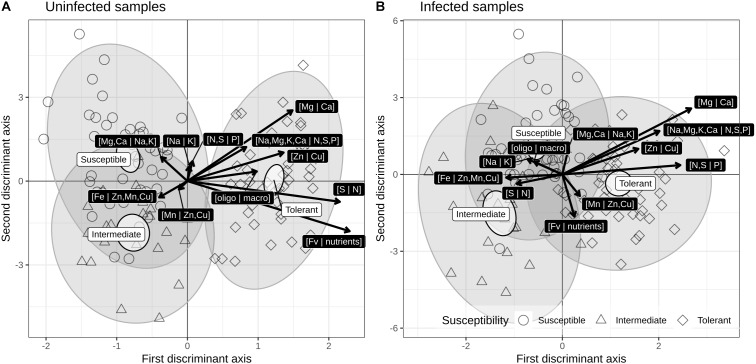
Correlation (scaling 2) biplots of linear discriminant analysis of susceptibility groups for **(A)** uninfected and **(B)** infected across BLS infection susceptibility groups of cultivars.

Discriminant analyses were used to show low-dimension swarms of infected and uninfected ionomes of susceptibility groups (susceptible, intermediate, tolerant). The resulting biplots (Figure 4A,B) show a slight difference due to infection, but significantly different means (non-overlapping 95% level ellipses around means). Susceptibility groups were not aligned through the susceptibility gradient, as would be the case if patterns were linear. While this could be due to non-linear patterns, this behavior could also result from imprecise susceptibility categorization. Nevertheless, tolerant cultivars were characterized by high [Mg | Ca], [Na,Mg,K,Ca | N,S,P], [N,S | P], [Zn | Cu], and [Fv | nutrients] balances and low [Fe | Zn,Mn] and [S | N] balances.

Interactions between infection and cultivars are in addition to infection and cultivar effects considered individually: they show how cultivars reacted differently to the infection as a difference between each interaction and the uninfected VIV cultivar. Here again, confidence intervals should be taken into consideration when evaluating pairwise differences. The most obvious interaction effects are observed for the infected PIC cultivar on balances related to N, S and P, i.e., [Na,Mg,K,Ca | N,S,P], [N,S | P] and [S | N]. There is no cultivar effect on the [Fv | nutrients] balance after infection with *Xcv*, except in the case of the HOC cultivar, which showed a slight but significant decrease. Moreover, no significant interaction effects on balances related to trace elements, i.e., [Fe | Zn,Mn,Cu], [Mn | Zn,Cu], and [Zn | Cu] were detected.

**Figure 5:**
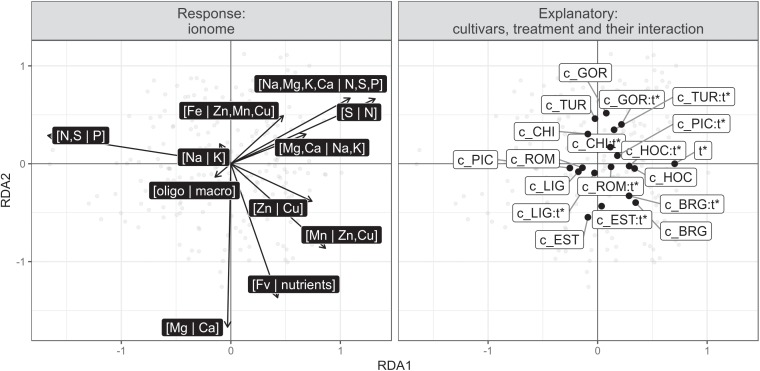
Redundancy analysis correlation triplot (scaling 2) with ionomic balances as response (dark labels) and cultivar, treatment and their interactions as descriptors. The reference case is the uninfected PIC cultivar, one of the susceptible cultivars chosen at random. c_ denotes cultivar, t^∗^ infected treatment and : (a colon) interaction.

These effects are consistent with the results of the redundancy analysis (RDA, Legendre and Legendre, 2012), which was used to map the effect of the infection on the ionome for each cultivar. The RDA was conducted with a response matrix previously detrended for random effects on nutrient balances (partial RDA conditioned with the experiment returned similar results), as well as features of cultivars and inoculation, and their interactions. The resulting triplot is presented in Figure 5.

The RDA summarizes the pattern of variation of the ionome according to the cultivar, the infection (treatment) and their interactions. The angles between vectors, both explanatory and response, reflect their correlation. The RDA confirmed the strong effect of infection on the [N,S | P] balance, where infection tended to shift the nutrient balance toward N,S, and away from to P. Indeed, the [N,S | P] arrow is pointing in the opposite direction from the treatment (t^∗^ and most of its interactions with cultivars, in Figure 5), indicating that [N,S | P] is negatively correlated with t^∗^ and most its interactions with cultivars. In a balance perspective, this means that treatment is associated with a decrease of P relatively to N and S – which is the equivalent of an increase of N and S relatively to P. Infection also tended to increase N relative to S, and anions relative to cations in macro elements, i.e., [Na,Mg,K,Ca | N,S,P]. With the exception of the BRG cultivar, all interaction scores are placed on the right of their cultivar scores, but behave heterogeneously on the vertical RDA axis, indicating discrepancies among cultivars in ionomic response to the infection, which is confirmed by the signifiance of the cultivar treatment interaction in the permutation test. Indeed, cultivar (*P* = 0.001), infection (*P* = 0.001), and their interactions (*P* = 0.001) were found to be significant at the 0.05 level.

### Effects of the Ionome and BLS Infection on Resistance

**Figure 6:**
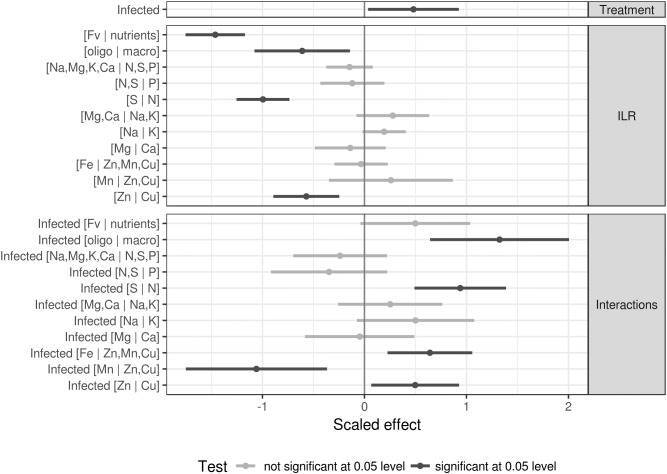
Linear effects of nutrient balances and infection on the severity index with experiment as random intercept. Significance was tested at the 0.05 level.

A linear mixed model was performed with the average disease severity index (Nicolas et al., 2018) as response and interacting treatment and centered and scaled ionomic balances as features, with the experiment as random intercept. Coefficients and the confidence intervals are shown in Figure 6.

It can be seen from Figure 6 that the infection itself increases the severity index. The severity index was reduced where nutrients loaded more than filling elements, as shown by the significant negative effect of the [Fv | nutrients] balance (significance tested at the 0.05 level). Significant negative trends were also observed for [trace elements | macro elements], [N | S], and [Zn | Cu] balances. Significant effects of similar magnitude were detected in the interactions.

The severity index of infected specimens was higher where trace elements decreased in the [oligoelements | macroelements] balance, where nitrogen increased relative to sulfur in the [S | N] balance and where copper increased relative to zinc in the [Zn | Cu] balance. The trends for these significant effects were opposite to those for their non-interacting counterparts. The [Fe | Zn,Mn,Cu] balance significantly increased the severity index of infected specimens, while this index decreased with increasing [Mn | Zn,Cu] balance.

## Discussion

The lettuce cultivars used in the present study have been characterized for their resistance to BLS by Nicolas et al. (2018). Data exploration revealed significant experimental effects of cultivar and BLS infection, as well as their interactions, on the ionome. Similar directions observed in the coefficients of cultivar and cultivar-treatment interactions could indicate that BLS infection can increase ionomic phenotype differentiation. All lettuce samples used for the mineral characterization were harvested 14 days after inoculation. Considering that lettuce nutritional value is strongly dependent on the growth stage and soil characteristics (Pinto et al., 2014) and that experiments were conducted in a greenhouse with variable temperature and insolation conditions, the discrepancies may be attributed to seasonal effects causing plants to acquire different nutrient balances depending on the specific growth conditions.

The approach proposed by Parent et al. (2013) focuses exclusively on balances between nutrients and does not consider the amounts of individual elements. The interpretation of nutrient balances, which is still uncommon in the field of plant science, offers robust statistics by mapping nutrients to multivariate coordinates as well as generating a sound collection of balances as univariate perspectives. Because elements act in a dependent manner, treating them independently can muddle thinking and interpretation (Baxter, 2015). Evidence that the elements of the ionome are interconnected is the identification of mutants with multi-element phenotypes reported particularly in *Arabidopsis* where many of these phenotypes have been shown to segregate as a single locus and quite a few have been cloned, verifying that a single polymorphism can affect multiple elements (Baxter, 2015). This dependence was recently reported by Zhang et al. (2017) who showed that Zn concentrations can affect nutrient transportation to tea leaves, leading to different distributions of foliar P, S, Al, Ca, Fe, and Cu.

Our study found that cultivars had notable ionomic trends, regardless of the treatment (Figure 3). The balances [Fv | nutrients], [Na,Mg,K,Ca | N,S,P], [N,S | P], and [Mg | Ca] differed markedly among the ionomes of uninfected cultivars. It is recognized that adequate intracellular concentrations of essential metal ions are required for both pathogen virulence and plant defense (Poschenrieder et al., 2006). Given the essential role of metals in living organisms, either a lack of, or an excess amount of, essential metals may have a profound effect on a wide range of pathogens and by extension, their interactions with the host (Fones and Preston, 2013). Compared to VIV, the [Fv | nutrients] balance differentiated tolerant cultivars from susceptible ones (Figure 3, 4). A positive [Fv | nutrients] value indicates that the geometric mean of nutrients is larger than the filling value. During plant-microbe interactions, high levels of metals can restrict pathogen colonization and be utilized as a defense mechanism by the host (Navarrete and De La Fuente, 2015). Plants absorb high concentrations of metals from the substrate as a self-defense mechanism against pathogens and herbivores (Poschenrieder et al., 2006). These findings are consistent with the results of our study in which tolerant cultivars showed [Fv | nutrient] values higher than those of intermediate and susceptible lettuce cultivars (Supplementary Table S2). A study conducted by Maity et al. (2016) on pomegranate (*Punica granatum* L.) found that concentrations of Ca, Mg, Mn, and Cu in leaves were significantly higher in germplasm with moderate resistance to bacterial blight disease compared to susceptible ones in which N, K, and S were observed to be high. However, in our study, high concentrations of mineral elements initially seemed to favor only tolerant lettuce cultivars despite a tendency toward ionome alteration in all cultivars after infection.

Considering that the lettuce cultivars were grown under identical conditions, differences in their ionome can be attributed to their genetics, naturally causing some of them to absorb more minerals than the others. Differences in the ionomes of plants growing under identical conditions reflect differences in their genomes and genes expression that have evolved through mutation and selection of adapted phenotypes (Neugebauer et al., 2018). A study conducted by Sánchez-Rodríguez et al. (2010) on tomato likewise showed significant differences in the nutrient concentrations of the cultivars even when their growing conditions were identical. Considering the role of nutrients in plant-pathogen interactions, it seems logical that high [Fv | nutrients] balances are found in cultivars with low susceptibility (Figure 6). Some plants, called “hyper-accumulators,” are known for their ability to absorb abnormally high levels of mineral elements from the soil which they store in their tissues (Fones and Preston, 2013). The most plausible hypothesis for the evolution of such plants is that hyper-accumulated metals provide a defense against pathogens (Poschenrieder et al., 2006). Although our data cannot be used to validate this hypothesis, they provide some support for it. High levels of metals can restrict host colonization by microbial pathogens and be utilized as a defense mechanism by the host during plant-microbe interactions (Navarrete and De La Fuente, 2015). This seems to be borne out by the results of our study, in which tolerant cultivars had the highest [Fv | nutrients] balance.

Our study showed that tolerant cultivars were characterized by high [Mg | Ca], [Na,Mg,K,Ca | N,S,P], [N,S | P], [Zn | Cu], and [Fv | nutrients] balances and low [Fe | Zn,Mn] and [S | N] balances (Figure 4). Two tolerant cultivars, HOC and BRG, also showed a positive [Mn | Zn,Cu] balance with a positive effect on infection with *Xcv* (Figure 3).

Calcium (Ca) is an important nutrient that affects susceptibility to diseases. Ca plays an important role in the stability of plant membranes and is an important component of the cell wall structure, as calcium galacturonates are required in the middle lamella for cell stability (Dordas, 2009). According to Marschner (1995), when Ca is deficient in a plant tissue, low-molecular-weight compounds such as sugars and amino acids leak through the membrane from the cytoplast to the apoplast, which stimulates infection by pathogens.

Micronutrients are required for the synthesis of secondary metabolites with antimicrobial activity (Poschenrieder et al., 2006). Copper (Cu) can restrict microbial colonization of the host. It is known that *Pseudomonas fluorescens* requires Cu detoxification genes to colonize its host (Zhang and Rainey, 2008). Also, high levels of zinc (Zn) are known to be deleterious for *X. fastidiosa*. Zn plays an important role in protein structure and starch synthesis and is involved in membrane protection from oxidative damage through the detoxification of superoxide radicals (Calmak, 2000). It is a structural or catalytic cofactor of many proteins (Navarrete and De La Fuente, 2015). Zinc is a cofactor for over 300 enzymes and 200 transcription factors associated with the maintenance of membrane integrity, auxin metabolism, and reproduction (Singh et al., 2016).

In the present study, we found that infection with *Xcv* mainly affected balances closely related to nitrogen (N) with a significant decrease in [N,S | P] and a significant increase in [Na,Mg,K,Ca | N,S,P] and [S | N] (Figure 3). A decrease in [N,S | P] balance with infection means that the concentration of N and S increases relative to that of P. Similarly, an increase in [Na,Mg,K,Ca | N,S,P] means that the concentration of N, S, and P increases with *Xcv* infection relative to that of Na, K, Mg, and Ca. Some essential nutrients have a greater impact on plant diseases than others. Generally, when N levels are deficient, plants are more susceptible to bacterial attack, whereas K and Ca play key roles in forming an effective barrier to infection (Bhaduri et al., 2014). The effect of N on disease severity depends on the type of pathogen. For obligate pathogens such as *Pseudomonas syringae*, low N concentrations lead to a decrease in severity of the disease (Hoffland et al., 2000), whereas the opposite effect is observed in the case of facultative pathogens such as *X. campestris* pv. *vesicatoria* (Chase, 1989) and some other *Xanthomonas* pathovars (Dordas, 2009).

The ability of a plant to withstand a pathogen depends primarily on the resistance factors involved or the capacity of the host to synthesize or mobilize defense factors. However, whether a plant is resistant or sensitive, it has the ability to initiate a defense reaction (Agrios, 2005) in an attempt to limit disease development. An increase in the N concentration in the host plant might be part of such a strategy to limit the spread of the pathogen.

Significant differences were found in all balances except [oligoelements | macroelements] for at least one uninfected cultivar (Figure 3). However, with the infection, only six balances showed significant differences. Although ionomic univariate differences can be detected in the cultivar-infection interactions, no significant effects emerged from five balances among our set, suggesting that there is some homogeneity in the ability of the host plant to mobilize nutrients after infection. This disappearance which was also observed in the [Fv | nutrient] balance confirmed the strong effect of *Xcv* infection on the ionome and can be explained by the ability of the host plant to mobilize nutrients after infection.

Because this study showed that tolerant cultivars collectively had a higher [Fv | nutrient] balance than susceptible and intermediate ones (Figure 3, 4), the increase of the [Fv | nutrient] balance with infection (Figure 3) could enhance the ionomic status of cultivars independently of their susceptibility. From a physiological standpoint, this means that overall lettuce tends to absorb more nutrients in response to infection. However, depending on the role of a specific element involved in defense, the plant will either increase or decrease the concentration of this element. Previous studies on other plant-pathogen interactions have found that modifications of the host leaf ionome occurred in response to pathogen attack (De La Fuente et al., 2013; Oliver et al., 2014). However, the change in the ionome which can be described as a nutritional immunity response depends on plant-pathogen interactions (Hood and Skaar, 2012). The present study provides additional evidence of this ability of plants to modify the ionome when infected.

There is evidence in the literature that plant disease symptoms can be associated with increased or unbalanced metal concentration in plant tissue (Fones and Preston, 2013). Kieu et al. (2012) showed that iron availability was the main factor limiting the infection of Arabidopsis by *Dickeya dadantii*. Also, data from Kieu et al. (2012) provided evidence that the disease intensity increased after bacterial infection on plants supplied with Fe compared to the iron-starved plants. According to Kieu et al. (2012), the plant iron status plays a key role on the expression of *D. dadantii* pectate lyase isoenzyme-encoding genes, pelA-pelD, the major determinants of symptom production on Arabidopsis. Our study showed that the [Fe | Zn,Mn,Cu] balance significantly increased the severity index of infected cultivars (Figure 6). With infection by *Xcv*, lettuce cultivars seem loading less amount of Fe, test significant at 0.005 level (Figure 6), to limit the availability of this element for the pathogen, another strategy that could be part of the nutritional immunity of lettuce.

With the analytical approach used in the present study, the effects of the ionome and BLS infection on resistance showed that infection increased the severity index (Figure 6). Figure 6 presents a model that can be used as a reference to establish proper management strategies for BLS using an ionomic approach. Four balances, [Fv | nutrients], [oligo | macro], [Zn | Cu], and [S | N], negatively affected the severity index. A preventive strategy based on suitable interpretation of these balances complemented with similar information could help in adjusting fertilization plans, thus enhancing the nutritional capacity of lettuce and increasing protection against BLS. This model can assist in the implementation of curative measures for better management of BLS and could also be extended to other plant-pathogen interactions.

This study showed that the lettuce ionome can be a relevant factor influencing the resistance of cultivars to BLS. Through their capacity to accumulate significant levels of minerals, tolerant cultivars are better able to limit pathogen damage compared to susceptible cultivars. Metal hyperaccumulation was reported by Kazemi-Dinan et al. (2015) as an elemental defense particularly in young plants. Infection of lettuce with *Xcv* resulted in changes in the ionome profile which enhanced cultivars’ resistance. Poschenrieder et al. (2006) used the expression “metal fortification” to describe this immune response strategy. Following on studies that have described resistance factors such as stomatal density (Nicolas et al., 2018), the present study shows the protective role played by the ionome in the interaction between lettuce and *Xcv*, which should be taken into consideration in lettuce fertilization and breeding programs. Research should be expanded to other crops interacting with plant pathogens in order to more thoroughly document the relationship between a given plant species’ resistance and its ionome. A multidisciplinary approach focusing on proteomics, transcriptomics, and metabolomics seems promising. Also, as reported in Huang and Salt (2016), a further study using the ionomics approach may allow the identification of genes involved in tolerance of lettuce to *Xcv*, and be used to design new cultivars with enhance resistance to BLS.

## Author Contributions

ON and VT conceived and designed the experiments and performed the sampling. ON and S-ÉP analyzed the data and wrote the manuscript, with the critical review of MTC, SJ, and CB. CB supervised the project. All authors approved the final version of the manuscript.

## Conflict of Interest Statement

The authors declare that the research was conducted in the absence of any commercial or financial relationships that could be construed as a potential conflict of interest.
