# Supplementary material for: The Ionomics of Lettuce Infected by Xanthomonas campestris pv. vitians
Source: Front Plant Sci. 2019 Mar 22;10:351. doi: 10.3389/fpls.2019.00351 (PMC6448033; doi:10.3389/fpls.2019.00351)
Supplement: Supplementary file 1 [file Table_1.docx]

Supplementary material for:

**The ionomics of lettuce infected by *Xanthomonas campestris* pv. *vitians***

**Olbert Nicolas^1,2^, Marie Thérèse Charles^2^, Sylvie Jenni^2^, Vicky Toussaint^2^, Serge-Étienne Parent^3^, and Carole Beaulieu^1^***

^1^Departement de biologie, Université de Sherbrooke, Sherbrooke, QC, Canada

^2^Agriculture and Agri-Food Canada, Saint-Jean-sur-Richelieu Research and Development Centre, Saint-Jean-sur-Richelieu, QC, Canada

^3^Department of Soils and Agri-Food Engineering, Université Laval, Quebec City, QC, Canada.

**Supplementary Table S1.** Ionome composition of lettuce cultivars used in this study, infected with *Xanthomonas campestris* pv. *vitians* and uninfected.

| **Cultivars** | **Nutrient composition ± S.E. (mg kg^-1^ dry tissue)** | | | | | | | | | | |
| --- | --- | --- | --- | --- | --- | --- | --- | --- | --- | --- | --- |
|  | Ca | Cu | Fe | K | Mg | Mn | Na | N | P | S | Zn |
| BRG inf. **^1^** | 11044 ± 1393 | 13.17 ± 4.47 | 73.8 ± 9.9 | 65154 ± 11784 | 3090 ± 265 | 123.7 ± 62.4 | 4590 ± 1524 | 42262 ± 865 | 6818 ± 1406 | 2773 ± 349 | 88.7 ± 8.6 |
| un. | 10610 ± 408 | 11.67 ± 0.95 | 105.2 ± 20.0 | 64777 ± 2498 | 3091 ± 95 | 128.2 ± 19.2 | 4167 ± 314 | 38049 ± 1872 | 7187 ± 384 | 2923 ± 72 | 83. ± 3.0 |
| CHI inf. | 8302 ± 1743 | 9.67 ± 3.63 | 53.0 ± 6.9 | 48594 ± 12207 | 2881 ± 192 | 130.9 ± 78.1 | 3372 ± 755 | 33448 ± 800 | 5381 ± 1232 | 2091 ± 419 | 65.7 ± 10.7 |
| un. | 8132 ± 325 | 6.92 ± 0.34 | 60.6 ± 5.0 | 46468 ± 4114 | 2978 ± 80 | 125.9 ± 21.3 | 3333 ± 294 | 31866 ± 1387 | 5972 ± 418 | 2132 ± 138 | 62.1 ± 1.7 |
| EST inf. | 13152 ± 1852 | 11.42 ± 3.73 | 69.3 ± 7.9 | 56345 ± 8417 | 3306 ± 306 | 118.0 ± 60.9 | 3773 ± 1199 | 37528 ± 2300 | 6253 ± 1269 | 2440 ± 230 | 80.3 ± 8.7 |
| un. | 12574 ± 379 | 9.17 ± 0.47 | 83.4 ± 9.0 | 57440 ± 1704 | 3250 ± 68 | 125.3 ± 17.1 | 3729 ± 250 | 31560 ± 1450 | 6625 ± 323 | 2416 ± 47 | 75.7 ± 1.8 |
| GOR inf. | 7725 ± 1137 | 8.42 ± 2.27 | 51.7 ± 7.0 | 51111 ± 10416 | 2798 ± 262 | 122.9 ± 64.8 | 2904 ± 755 | 36067 ± 1031 | 5960 ± 1313 | 1941 ± 459 | 68.2 ± 6.9 |
| un. | 7887 ± 385 | 8.25 ± 0.41 | 59.9 ± 3.3 | 54252 ± 1926 | 2769 ± 58 | 126.3 ± 19.6 | 2648 ± 236 | 28228 ± 1084 | 6473 ± 358 | 2053 ± 107 | 70.7 ± 1.9 |
| HOC inf. | 8869 ± 2212 | 11.33 ± 3.96 | 66.4 ± 8.9 | 51269 ± 10679 | 2797 ± 273 | 112.7 ± 59.1 | 3570 ± 972 | 36244 ± 734 | 5508 ± 1167 | 2185 ± 301 | 74.7 ± 11.0 |
| un. | 9148 ± 406 | 10.50 ± 0.70 | 69.4 ± 5.1 | 57340 ± 2812 | 2954 ± 81 | 102.9 ± 13.8 | 3573 ± 286 | 38969 ± 1311 | 6405 ± 499 | 2328 ± 143 | 74.2 ± 2.8 |
| LIG inf. | 11782 ± 1457 | 10.58 ± 2.78 | 72.8 ± 14.5 | 63410 ± 9495 | 3600 ± 188 | 154.5 ± 88.4 | 3397 ± 921 | 39788 ± 1402 | 7521 ± 1180 | 2413 ± 214 | 81.2 ± 9.1 |
| un. | 10982 ± 621 | 9.17 ± 0.53 | 73.6 ± 8.6 | 60964 ± 1881 | 3338 ± 90 | 141.3 ± 23.8 | 3180 ± 279 | 40080 ± 1835 | 7534 ± 351 | 2283 ± 165 | 75.7 ± 3.7 |
| PIC inf. | 9736 ± 1361 | 9.08 ± 3.55 | 47.7 ± 7.8 | 47289 ± 9260 | 2819 ± 278 | 118.0 ± 62.6 | 3252 ± 759 | 35387 ± 984 | 5478 ± 1069 | 1825 ± 390 | 58.6 ± 4.8 |
| un. | 10204 ± 430 | 8.50 ± 0.48 | 60.2 ± 7.0 | 47982 ± 1694 | 2822 ± 64 | 129.2 ± 22.0 | 3176 ± 220 | 25828 ± 2465 | 5993 ± 338 | 1973 ± 107 | 61.6 ± 1.6 |
| ROM inf. | 10629 ± 2050 | 10.33 ± 3.87 | 53.6 ± 7.0 | 54931 ± 13863 | 3521 ± 210 | 116.5 ± 64.6 | 3995 ± 1274 | 37356 ± 1354 | 6458 ± 1630 | 2284 ± 279 | 79.3 ± 11.1 |
| un. | 10168 ± 455 | 7.92 ± 0.50 | 75.7 ± 9.4 | 50020 ± 5296 | 3444 ± 65 | 118.2 ± 17.8 | 4002 ± 215 | 29628 ± 1412 | 7018 ± 397 | 2300 ± 87 | 74.0 ± 2.8 |
| TUR inf. | 7913 ± 2250 | 9.00 ± 2.83 | 50.0 ± 9.2 | 48441 ± 10471 | 2847 ± 267 | 135.0 ± 77.2 | 3393 ± 1096 | 32732 ± 1115 | 5323 ± 1063 | 1843 ± 411 | 65.8 ± 8.0 |
| un. | 7653 ± 557 | 7.67 ± 0.45 | 57.1 ± 5.2 | 46820 ± 1567 | 2840 ± 73 | 121.2 ± 19.1 | 3309 ± 386 | 26561 ± 1272 | 5604 ± 275 | 1880 ± 65 | 64.5 ± 1.7 |
| VIV inf. | 10884 ± 1817 | 10.58 ± 4.17 | 61.9 ± 8.2 | 58974 ± 11065 | 2968 ± 271 | 128.5 ± 61.7 | 3540 ± 1096 | 38691 ± 1901 | 5942 ± 1145 | 2465 ± 318 | 81.8 ± 12.6 |
| un. | 10596 ± 475 | 8.25 ± 0.41 | 64.3 ± 2.7 | 56895 ± 2363 | 2937 ± 75 | 128.7 ± 19.0 | 3640 ± 267 | 35464 ± 2240 | 6253 ± 361 | 2495 ± 96 | 75.2 ± 1.3 |

^1^inf.: infected, un.: uninfected

**Supplementary Table S2.** Mineral concentration in infected and uninfected lettuce cultivars according to their group of resistance to BSL (mg kg^-1^ dry tissue).

| **Resistance group^1^** | **Nutrient composition ± S.E. (mg kg^-1^ dry tissue)** | | | | | | | | | | |
| --- | --- | --- | --- | --- | --- | --- | --- | --- | --- | --- | --- |
|  | Ca | Cu | Fe | K | Mg | Mn | Na | N | P | S | Zn |
| TOLER. inf.^2^ | 11212 ± 1729 | 11.63 ± 3.73 | 70.6 ± 10.3 | 59044 ± 10094 | 3198 ± 258 | 127.2 ± 67.7 | 3833 ± 1154 | 38955 ± 1325 | 6528 ± 1255 | 2453 ± 274 | 81.2 ± 9.3 |
| un. | 10828 ± 453 | 10.13 ± 0.66 | 82.9 ± 10.7 | 60130 ± 2224 | 3158 ± 84 | 124.5 ± 18.5 | 3662 ± 282 | 37164 ± 1617 | 6938 ± 389 | 2488 ± 107 | 77.2 ± 2.9 |
| INTER. inf. | 9271 ± 2150 | 9.665 ± 3.35 | 51.8 ± 8.1 | 51686 ± 12167 | 3184 ± 238 | 125.7 ± 71.0 | 3694 ± 1185 | 35044 ± 1234 | 5890 ± 1347 | 2063 ± 345 | 72.6 ± 9.6 |
| un. | 8910 ± 506 | 7.80 ± 0.47 | 66.4 ± 7.3 | 48420 ± 3431 | 3142 ± 69 | 119.7 ± 18.4 | 3656 ± 300 | 28094 ± 1342 | 6311 ± 336 | 2090 ± 76 | 69.2 ± 2.3 |
| SUSC. inf. | 9162 ± 1515 | 9.43 ± 3.40 | 53.6 ± 7.5 | 51492 ± 10737 | 2866 ± 251 | 125.1 ± 66.8 | 3267 ± 841 | 35898 ± 1179 | 5690 ± 1190 | 2081 ± 397 | 68.6 ± 8.7 |
| un. | 9205 ± 403 | 7.98 ± 0.41 | 61.2 ± 4.5 | 51399 ± 2524 | 2876 ± 69 | 127.5 ± 20.5 | 3199 ± 254 | 30346 ± 1794 | 6173 ± 369 | 2163 ± 112 | 67.4 ± 1.6 |

*^1^TOLER.: tolerant group, INTER.: intermediate group and SUSC.: susceptible group.*

*^2^inf.: infected, un.: uninfected*
